# Vitreoretinal surgery: an introduction to simulation training

**Published:** 2023-12-01

**Authors:** James Rice, Jonel Steffan

**Affiliations:** Vitreoretinal Consultant: University of Cape Town Division of Ophthalmology, Cape Town South Africa.

**Simulation training can support vitrectomy surgery trainees to develop their skills and build a strong foundation of knowledge and understanding.**

Pars plana vitrectomy surgery requires a prolonged period of learning and mentorship and should not be performed without the necessary training and hands-on experience, some of which can be gained through simulation training. A virtual reality (VR) simulator, although expensive, can be a helpful tool for learning techniques; some, such as posterior hyaloid separation, is difficult to simulate in any other way. We recommend choosing a VR simulator that gives direct and immediate feedback and comes with multiple dexterity and navigation modules.

In the absence of a VR simulator, it is still possible to practise vitrectomy skills in more affordable ways.^1,3^ It is important to practise in as realistic a setting as possible: in the operating room used for retinal surgery and while using the operating microscope with indirect viewing system, the operating chair and microscope foot pedal, and resterilised retinal instruments.

## The indirect viewing system

**Figure 1:**
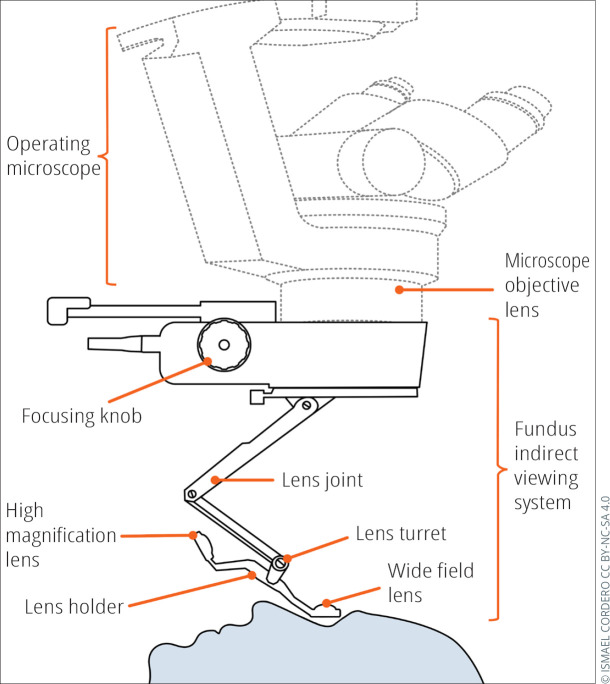
An indirect viewing system with automated inverter in the microscope.

Vitrectomy is performed using an indirect viewing system (Figure 1). This generates a wide-field, inverted view of the retina which is corrected with a prism inverter.

### How to use the indirect viewing system

Use the microscope controls to move the indirect viewing system closer to or further away from the eye. This controls the field of view. The closer the lens is to the cornea, the wider the field of view. The lens should be about 5–10 mm from the cornea. If it is too close, the lens may steam up. If it touches the cornea, it needs to be wiped dry.

To focus the image, rotate the focusing knob or dial on the indirect viewing system to alter the distance between its lenses. More advanced systems have electronics attached to the foot switch which can control this.

**Note:** viewing the retina through the indirect viewing system is very sensitive to eye rotation during surgery. Learn to instinctively adjust the microscope's X-Y joystick as you rotate the eye to view different parts of the retina. Move the microscope in the same direction as you rotate the eye, but be careful not to move too far or you will lose the optimal view.

## Tools for learning & practicing

### 1. Navigation, with rotation of the eye to view different parts of the retina

To practice navigation, we recommend making the simple model described in a previous issue of this journal (Figure 2a)^1^ but make the pupil diameter very small, as shown in Figure 2b. This helps to simulate the dexterity needed to maintain the view during eye rotation, as it requires very accurate microscope adjustments to maintain the view.

**Figure 2a:**
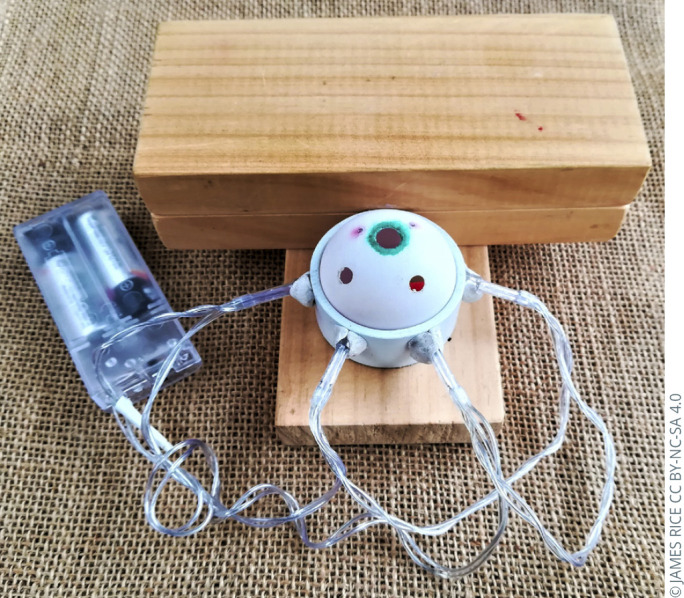
A simple model for practising tasks and navigation.

**Figure 2b:**
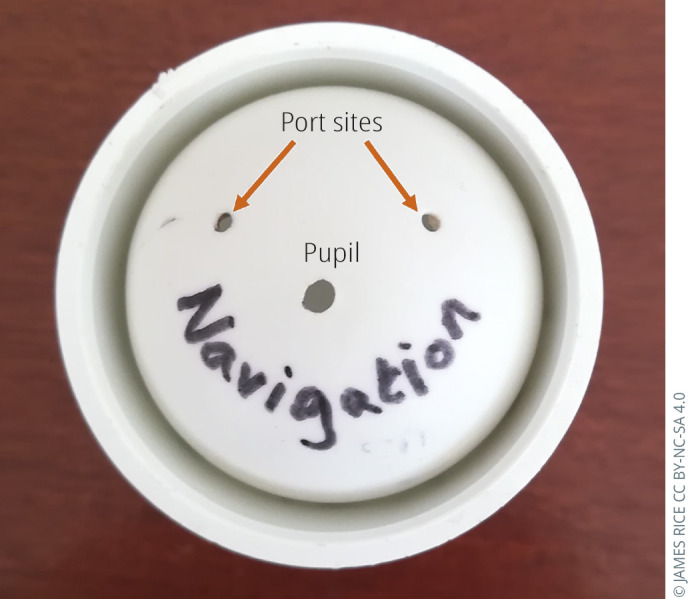
Model eye for practising navigation during simulated vitrectomy surgery.

### 2. Trocar insertion and infusion placement

Practise the following on soft silicone eyes. To keep costs in check, we work on eyes previously used for cataract surgery simulation.

**Figure 3:**
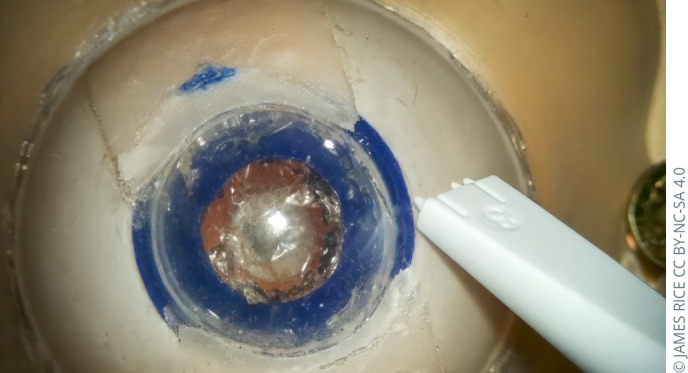
Measuring the position for insertion of the trocar.

**Figures 4a and 4b:**
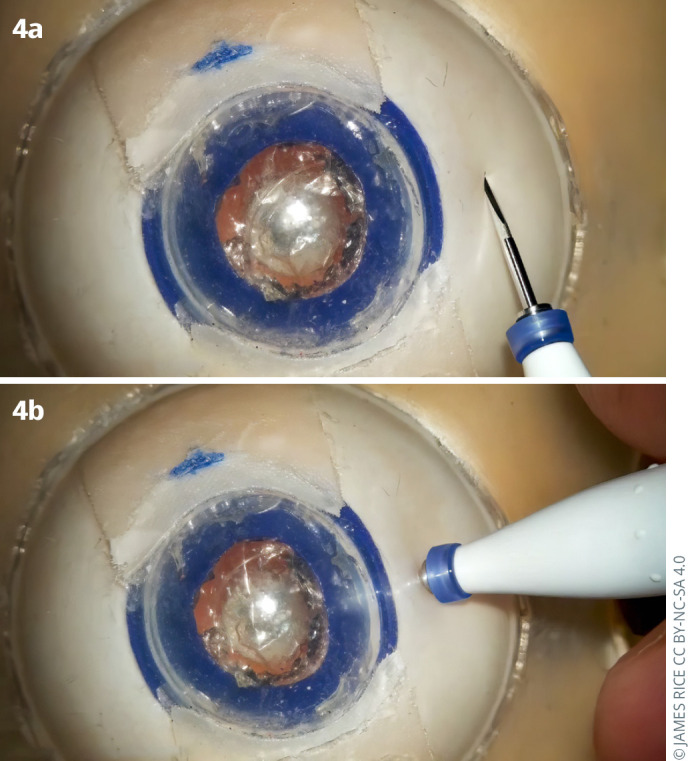
Two-step angled (or bevelled) insertion of the trocar.

- Measuring the position for inserting the trocars (Figure 3)
- The two-step angled (or bevelled) insertion of trocars (Figures 4b and 4c)
- Attachment of the infusion line.

### 3. Instrument insertion

Practice instrument insertion, usually with the vitrectomy probe and light pipe, while being aware of the correct angle or direction of insertion.

**Figure 5:**
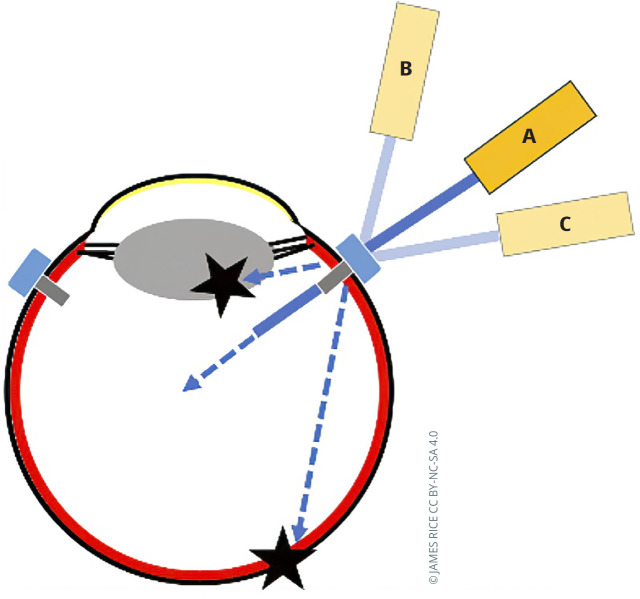
Position A is the correct angle for instrument insertion. B may result in retinal injury, and C in lens injury.

**Warning:** this process may result in retinal injury or lens injury if the angle of insertion is wrong (Figure 5).

### 4. Core vitrectomy and air fluid exchange

We practice core vitrectomy on specially designed artificial eyes, which we fill with egg white and ‘stain’ with triamcinolone acetonide. In the absence of a posterior segment machine, we use a 23-gauge anterior vitrectomy handpiece from the phaco machine and a separate light box.

**Figure 6:**
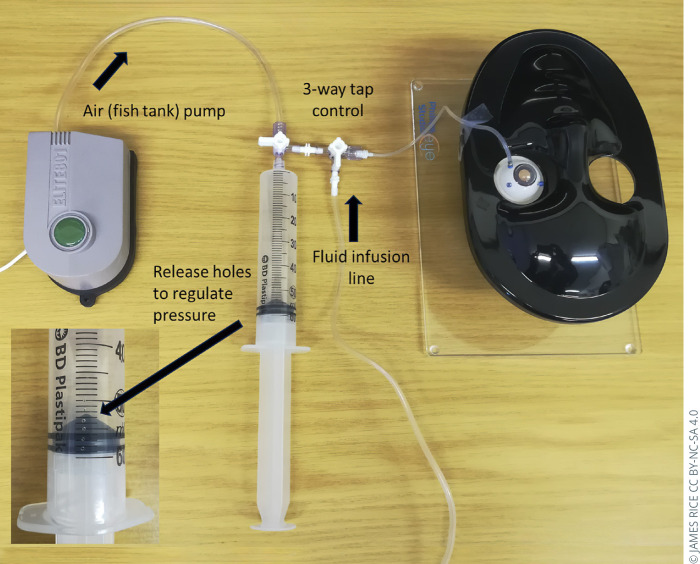
Infusion setup for air-fluid exchange.

By adding a 3-way tap and an air pump (a fish tank pump) we can practice core vitrectomy, blue dye stain of the epiretinal membrane, epiretinal membrane peel, air fluid exchange, and simulated gas injection. See Figure 6.

### 5. Indentation techniques

**Figure 7:**
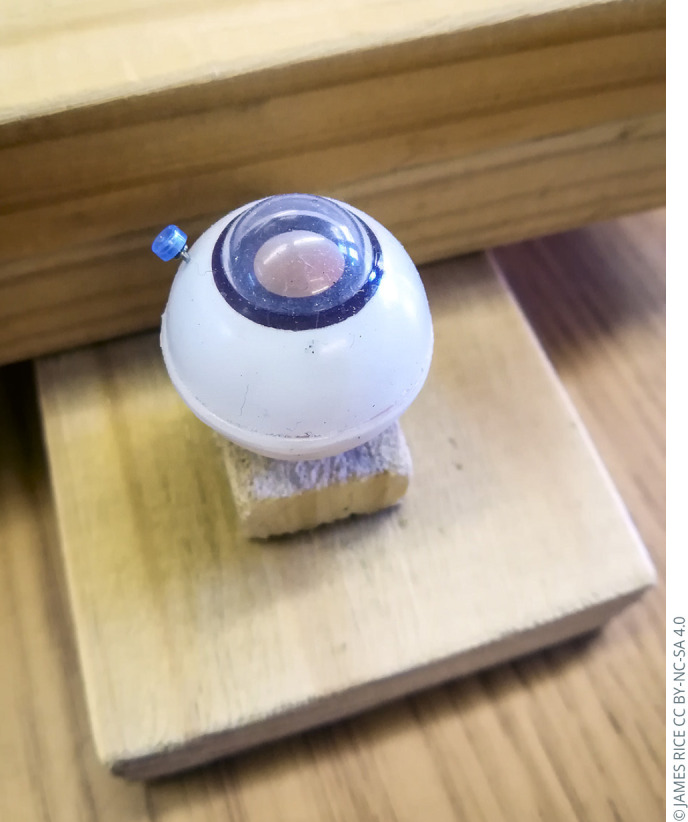
Mounting of a modified artificial eye, so it can be used for practicing indentation.

**Figures 8a and 8b:**
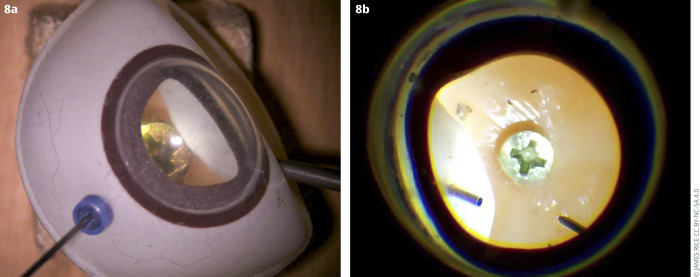
Direct view (a) and indirect view (b) of simulated, indented peripheral retina.

We recommend using a compressible artificial eye supported on a screw at the posterior pole which allows free rotation (Figure 7). This is useful for practicing a direct view of the indent (through the microscope only, Figure 8a) and an indirect view (through the indirect viewing system) (Figure 8b). You can also practice indented, ‘shaving’ manoeuvres if a chandelier light is available.

### 6. Dexterity tasks

It is helpful to learn to use retinal instruments by performing delicate manoeuvres under the indirect viewing system. You can practice these manoeuvres on affordable plastic eyes. Use real retinal forceps and scissors of various designs to become familiar with their squeezing action and practice bimanual techniques, which are usually the most challenging. Refer to the model described in a previous issue of this journal.^1^

## Tips for training others

Trainees should be taught how to set up a vitrectomy machine, as they may not have a skilled nurse to assist. They should also have an understanding of the effects of different settings. For example, a low cut rate removes the core vitreous more quickly than a high cut rate, but a high cut rate is safer when working near the retina. The different models of machines have significantly different settings and capabilities, and trainees need to be familiar with the machine they will use.

We recommend breaking vitrectomy surgery into individual surgical steps. Teach trainees details of the relevant ocular anatomy and pathology relevant to each step. Discuss surgical instrument design, handling, and the goals of the step, so that trainees have a detailed understanding of how to perform it accurately. Then demonstrate the step a few times, while giving a detailed explanation. Trainees should then perform the step and explain their actions. This is followed by sustained, deliberate practice, performing the step repeatedly. Trainers should observe and give feedback.

“We recommend breaking vitrectomy surgery into individual surgical steps. Teach trainees details of the relevant ocular anatomy and pathology relevant to each step.”

We provide trainees with written, descriptive guidelines for each step, which they refer to as they practice. Structured feedback and assessment tools, such as the Ophthalmology Surgical Competency Assessment Rubric for Vitrectomy,^4^ are available for live vitrectomy surgery. They are not fully transferrable to the simulation lab, but we still find them useful.^4^

Next, trainees should combine the steps to perform more complete procedures on a virtual reality simulator or on plastic eyes, as described above. Observe and give feedback and encourage the trainees to reflect on their performance. If video equipment is available, trainees should record and critically review their performance, preferably with the trainer.
